# Superb Micro-vascular Imaging (SMI): a Doppler ultrasound technique with potential to identify, classify, and follow up endoleaks in patients after Endovascular Aneurysm Repair (EVAR)

**DOI:** 10.1007/s00261-018-1633-x

**Published:** 2018-06-06

**Authors:** Marcin Gabriel, Jolanta Tomczak, Magdalena Snoch-Ziółkiewicz, Łukasz Dzieciuchowicz, Ewa Strauss, Katarzyna Pawlaczyk, Dorota Wojtusik, Grzegorz Oszkinis

**Affiliations:** 1 Department of Vascular and Endovascular Surgery, Angiology and Phlebology, Poznan University of Medical Sciences, Poznan, Poland; 2 Department of General and Vascular Surgery, Poznan University of Medical Sciences, ul. Długa ½, 60-848 Poznan, Poland; 3 The Lord’s Transfiguration Clinical Hospital, Poznan, Poland; 4 Institute of Human Genetics, Polish Academy of Sciences, Poznan, Poland; 5 Department of Hypertensiology, Angiology and Internal Diseases, Poznan University of Medical Sciences, Poznan, Poland

**Keywords:** Abdominal aortic aneurysm, Endovascular abdominal aortic aneurysm repair, Endoleak, Ultrasound, Superb Micro-vascular Imaging

## Abstract

**Purpose:**

The aim of the study was to assess the effectiveness of Superb Micro-vascular Imaging (SMI) as an alternative to Contrast-Enhanced Ultrasound (CEUS) and Computed Tomography Angiography (CTA) for endoleak detection and classification in patients followed up after endovascular abdominal aortic aneurysm repair (EVAR).

**Materials and methods:**

From May 2015 to January 2017, 30 patients underwent post-EVAR follow-up with Color Doppler Ultrasound (CDUS), CEUS, SMI, and CTA examinations. Aneurysmal sac diameter and graft patency were evaluated; endoleaks were identified and classified. Sensitivity, specificity, and accuracy values were calculated for each of the four diagnostic methods of endoleak detection. A percentage of agreement and Cohen’s Kappa coefficient were calculated for comparison of methods in terms of endoleak identification.

**Results:**

CTA revealed fifteen endoleaks (50%): three type Ia, nine type II, and three type III. The sensitivity of CDUS, CEUS, and SMI relative to CTA was 27%, 100%, and 100%, respectively. Specificity was 93%, 93%, and 93%, respectively. Accuracy was 60%, 97%, and 97%, respectively. There were no differences between SMI and CEUS in terms of sensitivity, specificity, or accuracy (100%, 93%, and 97%). We do not observe statistically significant differences between CTA, CEUS, and SMI concerning endoleak identification ability. The weakest method in endoleak identification was CDUS.

**Conclusions:**

The analysis showed that SMI is effective, repeatable, and comparable with the CEUS modality in identification endoleaks after EVAR; it may be considered as a potential tool to monitor patients after EVAR implantation, especially those with renal insufficiency or with an allergy to any contrast media.

Patients after Endovascular Aneurysm Repair (EVAR) require regular and strict follow-up to enable early detection of endoleaks and their treatment. The use of Computed Tomography Angiography (CTA), which is currently recommended for the follow-up of patients undergoing EVAR, puts the patients at risk of receiving high doses of ionizing radiation and exposes them to the repetitive impact of nephrotoxic X-ray contrast [1, 2].

Another method used for EVAR follow-up is ultrasound, which is a well-established and non-invasive diagnostic tool with real-time tissue harmonic imaging enabling diagnosis and prompt implementation of a treatment plan; it is particularly useful in vascular imaging where precise hemodynamic data are decisive [3–5]. Endoleaks in Color Doppler Ultrasound (CDUS) appear as pulsatile color flow within or adjacent to the aneurysm sac [2]. Some researchers suggest that CDUS can be used for the follow-up of patients after EVAR [6–8]. In turn, other authors state that CDUS alone is insufficient for endoleak identification in routine clinical practice [9–11].

High-resolution contrast-enhanced ultrasound (CEUS) is one of the alternative methods used to detect and characterize endoleaks [1, 2]. The advent of CEUS imaging utilizing sulfur hexafluoride microbubbles as a contrast agent improved the resolution and sensitivity of microvessel visualization. Moreover, the rate of adverse reactions reported for microbubbles is low, and no contrast-induced effects on renal function have been observed [2]; notwithstanding, the method’s requirement of intravenous contrast agents remains a disadvantage.

Recently, Toshiba has developed an innovative Doppler ultrasound technology called Superb Micro-vascular Imaging (SMI) using the Aplio™ 500 ultrasound system (Toshiba Medical Systems Corporation, Tochigi, Japan), which enables the visualization of slow-flow vessels without the need to use a contrast medium; it appears to be a new promising tool for the detection of endoleaks [3–5]. This advanced method offers unique advantages, including low-velocity flow visualization, high-resolution imaging, minimal motion artifacts, and high frame rates. The technique employs a proprietary adaptive algorithm to remove clutter artifacts while maintaining sensitivity to low blood flow velocities [3, 13]. SMI is able to visualize lower-velocity blood flows without the negative influence of motion artifacts arising from nearby structures, which is impossible to achieve with conventional Doppler techniques [13]. SMI can rapidly confirm blood flow or detect the absence of flow in cases of torsion and ischemia [3, 12]. SMI works in two modes: the monochrome (grayscale) mode (mSMI), which improves sensitivity by subtracting the background information and focusing only on the vasculature, and the color mode (cSMI), which demonstrates B-mode and color information simultaneously [3, 4, 13].

The aim of the present study was to evaluate the effectiveness of SMI as an alternative to CDUS, CEUS, and CTA in the identification and classification of endoleaks in patients undergoing post-EVAR follow-up, using CTA as the reference method.

## Materials and methods

The study prospectively analyzed 30 patients after EVAR for abdominal aortic aneurysm or iliac aneurysms. All procedures were performed at the Department of General and Vascular Surgery of the Poznan University of Medical Sciences in the years 2011-2016. CDUS, CEUS, SMI, and CTA examinations were performed between May 2015 and January 2017. Our institutional post-EVAR protocol consisted of CTA one month after repair, followed by CDUS after 6 months, and then CDUS once a year. The study was carried out in accordance to the Declaration of Helsinki, and its protocol was approved by the Bioethical Committee of the Poznan University of Medical Sciences (decision no. 963/12).

The indications to use CEUS and SMI were as follows:

1. Unclear CDUS results during routine follow-up; CEUS and SMI were used in order to verify the results and characterize the endoleak type.
2. Possible endoleak existence suspected intraoperatively during the EVAR implantation.
3. Assessing the outcome of the secondary intervention after endoleak diagnosis.
4. Aneurysm expansion without an identifiable endoleak on CDUS.

Patients with inability to keep still were excluded from the study.

The CDUS, SMI, and CEUS examinations were performed (in this order) by an experienced vascular surgeon blinded to the results of CTA, using Toshiba’s Aplio™ 500 device equipped with a 4–6-MHz curved array transducer. Moreover, two additional examiners (a vascular surgeon and a radiologist) independently assessed the video and images. The initial examination was a standard morphological investigation in 2D presentation (B-mode) followed by blood flow analysis using CDUS. Subsequently, cSMI and mSMI examinations were conducted. Finally, CEUS was performed after the administration of an intravenous 2.5-milliliter bolus of ultrasound contrast—SonoVue (Bracco, Milan, Italy); the duration of the CEUS examination is 2–3 min. CTA was performed using a GE 64 VCT Lightspeed multislice angiography scanner (acquisition 64 × 0.625 mm, helical mode, tube voltage 80e140 kV, tube current 300 mAs, CTDI Vol 14 mGy, rotation time 0.32, pitch 0.7, slice collimation 0.6, slice width 1.5 mm in retro-reconstruction) [4]. CTA was performed no later than 2–3 weeks after the CDUS, CEUS, and SMI examinations and was used as the reference method.

The findings of all four imaging modalities were recorded, including the presence of endoleaks and their types, the types of the endoleaks’ inflow vessels, and the patency of the stent-graft segments.

The maximal aneurysm sac diameter was measured from the leading edge of the anterior wall to the leading edge of the posterior wall on the transverse plane. Continuous data are presented as the mean ± standard deviation (SD) or median [interquartile range (IQR)] as appropriate; qualitative parameters were expressed as percentages. Sensitivity, specificity, and accuracy values were estimated for all imaging modalities. To compare the methods in terms of endoleak identification, the percentage of agreement and Cohen’s Kappa coefficient were calculated. The differences in endoleak identification between the studied methods were considered statistically significant when the observed *P* value for proportionate agreement was < 0.05. The level of statistical significance has been determined using the *χ*^*2*^ test or Fisher’s exact test and GraphPad Prism software (version 6.04).

## Results

### Patient characteristics

This prospective study included 30 patients (27 males and 3 females) at the mean age of 70.1 years (SD ± 10.3) who had undergone EVAR procedures; the mean aneurysm diameter was 6.41 cm (SD ± 1.9, range 3.8 –10.5 cm). Patient characteristics are presented in Table 1.

**Table 1:** Patient characteristics

Patient characteristics *n* = 30	Percentage
Smoking (present)	36.7
Obesity BMI > 30 [(kg/m^2^)	40.0
Diabetes	36.7
Hypertension	83.3
Hyperlipidemia	30.0
Coronary artery disease	63.3
Renal insufficiency	23.3

Variables expressed as %

The median period of time between the procedure and the imaging studies was 165 days (IQR 52, 839). The analyzed patients underwent different EVAR procedures. In 24 patients, EVAR implantation was performed to treat infrarenal abdominal aortic aneurysms (23 bifurcated stent grafts and one Nellix device were implanted). Another three patients had isolated common iliac artery aneurysms and were implanted with uni-iliac devices. Finally, three patients were treated with fenestrated endografts (using from 1 up to 4 branches). The procedures were performed with the following devices: Zenith (Cook Medical, Bloomington, IN, USA): *n* = 21; Excluder (W.L. Gore and Associates, Flagstaff, AZ, USA): *n* = 6; E-vita (JOTEC, Hechingen, Germany): *n* = 2, and Nellix (Endologix, Irvine, CA, USA): *n* = 1. The choice of the specific stent graft depended on the morphology of the abdominal aortic aneurysm and was made in accordance with the manufacturer’s guidelines in all cases.

### Endoleak identification

During the B-mode examination, the correct location of the stent graft and preserved continuity of its walls were clearly visible in all studied patients. No stent-graft deformation was observed. In all cases, CDUS, SMI, and CEUS revealed the presence of blood flow in the stent-graft lumen. We did not observe any adverse reactions or complications during the use of either CTA or the microbubble contrast agent.

**Fig. 1:**
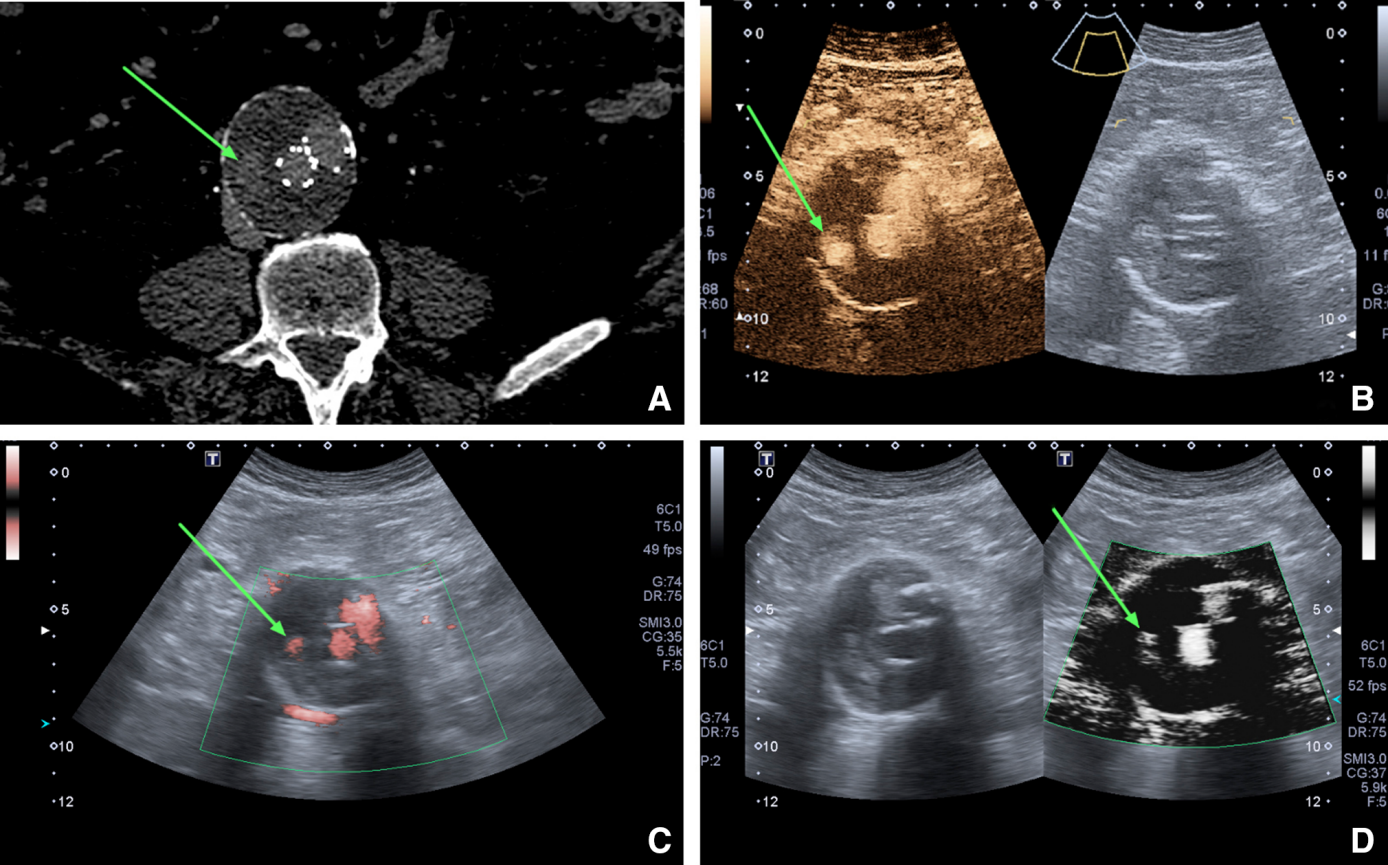
Cross section through an abdominal aortic aneurysm showing a type IIb endoleak (green arrow). **A** Computed Tomography Angiography, **B** Contrast-Enhanced Ultrasound, **C** color Superb Micro-vascular Imaging; **D** monochrome Superb Micro-vascular Imaging

**Fig. 2:**
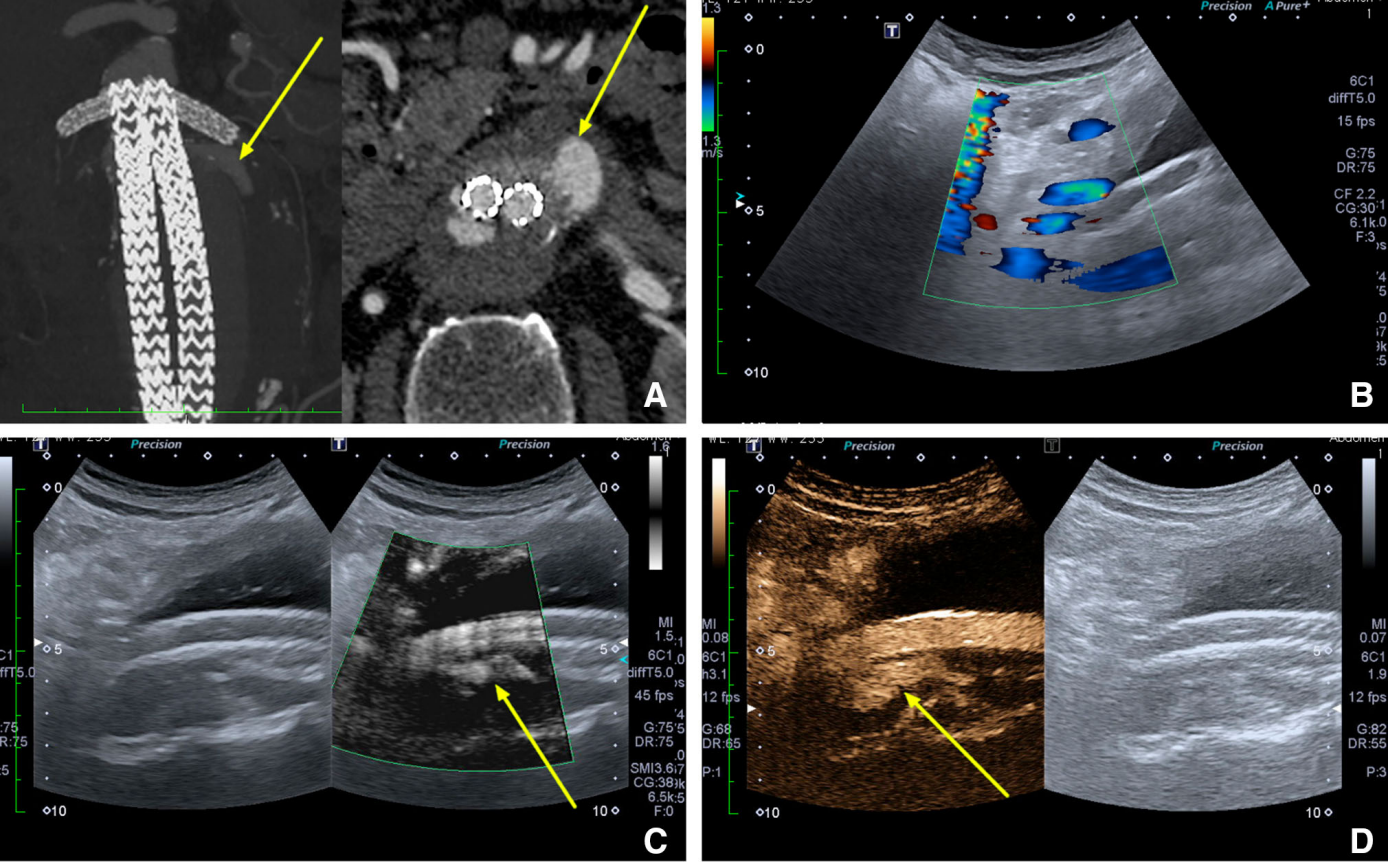
A type I endoleak (yellow arrow) according to four different imaging methods of an abdominal aortic aneurysm: **A** Computed Tomography Angiography, **B** Color Doppler Ultrasound, **C** monochrome Superb Micro-vascular Imaging, **D** Contrast-Enhanced Ultrasound

CDUS identified endoleaks in 17% of the enrolled patients (5/30 cases), CEUS and SMI in 53% of patients (16/30 cases), and CTA (the gold standard) identified endoleaks in 50% of patients (15/30 cases). The majority of the identified endoleaks were type II (Fig. 1), then three type Ia (Fig. 2), and three type III. No type IV or type V (endotension) endoleaks were diagnosed in the study group.

The detailed findings were as follows. Fifteen endoleaks were correctly detected by CEUS and SMI as well as CTA. One additional endoleak was detected by both CEUS and SMI; however, it was not confirmed by CTA (the gold standard). This means that CEUS and SMI yielded only one false-positive result each. All endoleaks identified by SMI were confirmed by both cSMI and mSMI. CDUS revealed only five endoleaks, and one of these results was a false positive; the method failed to detect endoleaks in as many as 11 patients. The number and types of the endoleaks are presented in Table 2.

**Table 2:** The number of endoleaks detected by each imaging modality

Variable	Imaging modality			
	CDUS	CEUS	SMI	CTA
No endoleak detected:	25	14	14	15
Endoleak detected:	5	16	16	15
I	0	3	3	3
Ia	0	3	3	3
Ib	0	0	0	0
II	5	10	10	9
IIa (IMA)	1	2	2	2
IIb (LA)	3	7	7	5
IIa + IIb	1	1	1	2
III	0	3	3	3

*CDUS* Color Doppler ultrasound, *CEUS* Contrast-Enhanced Ultrasound, *SMI* Superb Micro-vascular Imaging (cSMI & mSMI), *CTA* Computed Tomography Angiography, *IMA* Inferior Mesenteric Artery, *LA* Lumbar Arteries

The sensitivity, specificity, and accuracy of SMI and CEUS were the same (100%, 93%, and 97%, respectively). The sensitivity, specificity, and accuracy of CDUS, CEUS, and SMI in identifying endoleaks are summarized in Table 3. It is worth noting that CEUS and SMI were distinctly more accurate than CDUS in the identification of endoleaks, and their accuracy was similar to CTA.

**Table 3:** Sensitivity, specificity, and accuracy of the analyzed imaging modalities in detecting post-EVAR endoleaks

Imaging modality	True positives	False positives	False negatives	True negatives	Sensitivity (%)	Specificity (%)	Accuracy (%)
CDUS	4	1	11	14	27	93	60
CEUS	15	1	0	14	100	93	97
SMI	15	1	0	14	100	93	97

*CDUS* Color Doppler Ultrasound, *CEUS* Contrast-Enhanced Ultrasound, *SMI* Superb Micro-vascular Imaging (cSMI & mSMI)

There were no statistically significant differences between CTA, CEUS, and SMI concerning their ability to identify endoleaks (Table 4). However, the results indicate that CDUS is the least reliable method in endoleak identification. We observed that the percentage of agreement between CDUS and CTA was only 66.7% (*P* = 0.006, Cohen’s Kappa coefficient was 0.333), and this proportion was similar when CDUS was compared to SMI/CEUS (63.3% at *P* = 0.003 and 0.477, respectively).

**Table 4:** Comparison of methods in terms of endoleak identification

Endoleak identification	SMI vs. CTA	CEUS vs. CTA	SMI vs. CEUS	CDUS vs. CTA	CDUS vs. SMI/CEUS
Agreement [%], *P* value	96.7, 0.796	96.7, 0.796	100.0, 1.000	66.7, 0.006	63.3, 0.003
Cohen’s Kappa coefficient	0.935	0.935	1.000	0.333	0.477

*CDUS* Color Doppler Ultrasound, *CEUS* Contrast-Enhanced Ultrasound, *SMI* Superb Micro-vascular Imaging (cSMI & mSMI), *CTA* Computed Tomography Angiography

## Discussion

EVAR procedures require close lifelong imaging surveillance to quickly detect any possible complications such as endoleaks, fractures, graft migration, kinking, or enlargement of the aneurysm sac with probable aneurysm rupture [2, 10].

CTA is currently considered to be the gold standard for monitoring during post-EVAR follow-up. CTA imaging is widespread and provides precise aneurysm sac measurements. Moreover, it enables rapid and reproducible acquisition of data (ensuring homogeneous results among institutions) with high diagnostic value. CTA also provides superior information related to the stent graft, such as patency, the presence of endoleaks and their type, anchoring, or kinking [10, 11].

Furthermore, a systematic review by Karthikesalingam et al. pointed out that the mean sensitivity and specificity of CTA in endoleak detection is 70% and 98%, respectively [14]. However, the method has a number of significant disadvantages. One example is the high and cumulative dose of ionizing radiation (more than 20 mGy per one thoraco-abdominal CT scan), which significantly increases the risk of cancer. Moreover, the nephrotoxic contrast agent load causes a significant decline in renal function during long-term EVAR monitoring. Finally, the method is associated with high costs. These drawbacks prompted the search for a diagnostic technique that would be better suited for long-term surveillance [10, 15–17]. Because of these factors, a safer and more cost-effective alternative to CTA is required [2, 14, 16].

CDUS is an inexpensive and non-invasive imaging modality with no risk of radiation exposure or contrast-induced renal insufficiency, and it has been, therefore, investigated as an alternative to CTA for post-EVAR monitoring [10]. On the other hand, CDUS has major limitations, including the notable rates of false-positive and false-negative results as well as significant operator dependency [18]. Additionally, CDUS imaging seems to be impeded by unfavorable body habitus (e.g., obesity), ascites, or excessive intervening bowel gas, as well as graft factors such as echo reflection from the stent graft, stent-graft failure, kinking of the limbs, or slow endoleak flow [9, 19].

In recent years, numerous studies and a meta-analysis have shown the increasing role of CEUS in post-EVAR surveillance. The results demonstrated that CEUS is highly diagnostic and comparable to CTA with regard to detecting and characterizing endoleaks; moreover, it is not associated with renal impairment or ionizing radiation. Additionally, CEUS is more cost-effective than CTA [2]. A systematic review by Karthikesalingam et al. emphasized that CEUS, with its specificity of 90%–97% and sensitivity of 62%–83%, is useful in endoleak detection [14]. In the present study, the specificity of CEUS in endoleak detection was 93%, while its sensitivity was 100%. Furthermore, in a meta-analysis published by Mirza et al., the authors confirmed that CDUS had insufficient sensitivity for endoleak detection and praised CEUS as a highly sensitive diagnostic tool [19]. On the other hand, CTA is unsuitable for detecting some types of slow endoleaks which can be visualized with CEUS, MRA [20–22], or, as we have shown in the present study, SMI.

Accordingly, several authors considered CEUS as the first imaging tool for post-EVAR surveillance as it appears to be more specific than CTA for low-flow endoleaks [10, 23]. Their results also indicate that CEUS enables better classification of endoleaks because of its ability to obtain real-time hemodynamic information about the direction of blood flow [10, 23], which is particularly helpful in planning redo procedures, especially in patients with, type II endoleaks [24].

The EFSUMB guidelines of 2011 recommend the use of CEUS in post-EVAR follow-up (level of evidence: 1a). The main obstacle for the widespread use of CEUS is that equipment for contrast imaging is not available in all centers [2]. Moreover, the time of examination is limited to only a few minutes, and extensive operator experience is required.

Both CEUS and SMI are vascular imaging methods that provide outstanding detectability and classification of endoleaks, visualizing them in real time. However, SMI provides continuous, real-time scanning without any time constraints. No intravenous contrast agent is needed with SMI, which is one reason why it is better suited for detecting or monitoring endoleaks in patients with renal failure. In particular, the fact that SMI can be used separately or in combination with contrast agents makes it a novel and promising tool for visualizing endoleaks [12, 13, 25]. SMI can also save time, increase sensitivity, and improve diagnostic accuracy as well as treatment planning. Compared with conventional Doppler techniques, SMI offers better detail resolution, faster frame rates, less clutter, and fewer flash artifacts [3, 12]. The existing Doppler modes are unable to distinguish motion artifacts from actual blood flow. With SMI, it is possible to analyze the characteristics of such motion artifacts and extract only the clinically relevant information [13].

In addition, the proper performance of an SMI examination requires several conditions to be met. During the scanning of individual vascular sections, the probe must be maintained in a stable position, without shifting or vibrating. When small endoleaks are examined, it may be helpful to ask the patient to temporarily refrain from breathing. It should be taken into account that, at least in some cases, the test is time-consuming. The presence of intestinal gases or increased peristaltic movements may result in the occurrence of artifacts that impede vascular assessment. The amount of gases in the examined area can be reduced by massaging the area with the probe to displace the gas to other abdominal regions. In the case of increased peristalsis, it may be helpful to change the position of the body or to take a few-minute break.

The first case report about the use of SMI for EVAR surveillance comes from our research team; we demonstrated promising results of using SMI for endoleak detection, comparing the method with CDUS, CEUS, and CTA [4]. Cantisani et al. compared SMI with CDUS, CEUS, and CTA. The authors examined 57 patients after EVAR procedures and confirmed the presence of 8 endoleaks (all type II). The study demonstrated that the sensitivity of CTA, CEUS, CDUS, and SMI was 88%, 100%, 63%, and 75%, respectively. According to the results, SMI was more accurate than CDUS, but less accurate than CEUS and CTA in identifying endoleaks. This led the authors to recommend SMI for post-EVAR follow-up, especially in cases when CEUS or CTA cannot be used [26]. The difference in the number of endoleaks reported by Cantisani et al. and our study may influence the difference in sensitivity in both studies (75% vs. 100%, respectively). Despite the large number of patients participating in the Cantisani et al. investigation, the authors have observed a small number of endoleaks. Moreover, they encountered only type II endoleak, which is the most frequent type of endoleak. Accordingly, not all types of endoleaks were represented in mentioned study. Contrary to the findings showed by Cantisani et al., in our study we observed type Ia, II, and III endoleaks.

The recent study presents the currently largest case series of endoleaks examined with CTA, SMI, and CEUS (*n* = 15), showing that the three modalities have nearly the same sensitivity, specificity, and accuracy in endoleak detection. In the present study, CEUS and SMI detected a higher number of endoleaks compared to CTA, yielding only one false-positive result each. As a reference method, CTA still allows for better classification of endoleaks compared to CEUS and SMI. However, the high sensitivity and specificity found in this study lead to the conclusion that CEUS and SMI may be equivalent to CTA, especially in long-term EVAR follow-up.

According to the still scant literature about the application of SMI, the method appears to have significant clinical value in terms of early diagnosis and treatment planning in patients with cancer and tumors, facilitating the evaluation of the shape and density of the tumor vessels. Other potential clinical applications of SMI include gynecological and obstetric imaging, evaluation of lymph nodes and abdominal areas, as well as diagnosis of skin lesions, rheumatoid arthritis, and many other medical conditions [4, 5, 13, 27, 28].

As is the case with CDUS, CEUS, and CTA, SMI has some disadvantages. The SMI technology available only uses the Toshiba ultrasound system. Besides the drawbacks mentioned in the earlier discussion of CDUS, a crucial issue related to SMI is that it requires substantial experience from the operator performing the examination. Like CEUS, SMI is an examination with high operator dependency and requires a learning curve. Additionally, interpreting SMI results is particularly difficult for inexperienced operators. Neither CEUS [10] nor SMI should be used as the only imaging technique for EVAR surveillance, particularly when reintervention is required. Nevertheless, these methods can certainly replace most CTA examinations and act as a first-line modality filtering which patients should undergo CTA if an endoleak is detected. Thus, the use of SMI and CEUS would significantly reduce the number of CTA examinations and, in consequence, decrease the load of ionizing radiation and the risk of renal insufficiency [10]. Moreover, the new Toshiba technology has the potential to supplement or replace CEUS and CTA for completion imaging to reduce the use of contrast media.

The limitation of the present study is the small sample size. A study involving more patients with endoleaks after EVAR is needed to confirm the results and to assess the differences in endoleak classification. The major obstacle for the use of the SMI modality for EVAR surveillance is that, at the moment, the only distributor of SMI equipment is Toshiba. Moreover, the SMI method is characterized by operator dependency, difficulties in interpreting the results, and, finally, the lack of a defined protocol.

## Conclusion

The study demonstrated that SMI was more accurate than CDUS, identically as accurate as CEUS, and slightly less accurate than CTA in detecting endoleaks after EVAR. SMI and CEUS had the same values of sensitivity, specificity, and accuracy, which indicates that they may be equivalent in endoleak detection. SMI is a new non-invasive method with potential application in endoleak detection during EVAR follow-up, without additional risk of ionizing radiation exposure, use of any contrast media, or nephrotoxicity. Therefore, SMI can be used repeatedly even in patients with renal insufficiency. In the near future, SMI can become an essential method for the diagnosis and assessment of endoleaks with an effectiveness comparable to CTA and CEUS.
